# Effect of Acute Caffeine Intake on Maximal Aerobic Speed in University Soccer Players Assessed by the 30–15 Intermittent Fitness Test

**DOI:** 10.3390/sports14030123

**Published:** 2026-03-23

**Authors:** Diego Camilo García-Chaves, Juan Pablo Fernandez Zapata, Tatiana Oyaga Álvarez, Nelson Ortiz Escobar, Alfonso Villegas Mazo, Luisa Fernanda Corredor-Serrano

**Affiliations:** 1 Specialization in Theory and Methodology of Sports Training, Faculty of Education and Sport Sciences, Institucion Universitaria Escuela Nacional del Deporte, Cali 760041, Colombia; toyaga@endeporte.edu.co (T.O.Á.); nelson.ortiz3@endeporte.edu.co (N.O.E.); luisa.corredor@endeporte.edu.co (L.F.C.-S.); 2 Institute of Sport, Physical Education and Recreation of Valle del Cauca, Sports Medicine Center, Cali 760041, Colombia; juanpafer1494@gmail.com; 3 Soccer Center Research Seedbed, Football Academic Chair, Institucion Universitaria Escuela Nacional del Deporte, Cali 760041, Colombia; avillegas@endeporte.edu.co

**Keywords:** caffeine, soccer, 30–15 IFT, maximal aerobic speed, intermittent performance

## Abstract

The aim of this study was to analyze the effect of acute caffeine intake on maximal aerobic speed (MAS) assessed using the 30–15 Intermittent Fitness Test (IFT) in university soccer players. An experimental, randomized, double-blind, crossover design was employed, involving 26 male university team players (*n* = 26). Each participant completed the test under two conditions: caffeine supplementation (220 mg; 2.85 ± 0.27 mg/kg, range 2.60–3.16 mg/kg) and placebo, separated by a 72 h washout period. The final running speed achieved (VIFT) was used as an estimator of MAS. Statistical analysis included descriptive statistics, normality testing, and paired Student’s *t*-test, with a significance level set at *p* < 0.05. The results revealed a significant improvement in VIFT under the caffeine condition (19.94 ± 1.67 km/h) compared with placebo (18.72 ± 1.50 km/h), with a mean difference of 1.22 km/h (6.5%) and a large effect size (dz = 1.24; *p* < 0.001). It is concluded that acute caffeine intake was associated with a significant improvement in intermittent aerobic performance in university soccer players under the conditions of the present study, suggesting that caffeine may represent a potentially useful strategy in similar applied contexts.

## 1. Introduction

Soccer is an intermittent sport that requires continuous interaction between aerobic and anaerobic energy systems, due to the alternation between high-intensity actions such as accelerations, decelerations, and changes in direction, combined with periods of active recovery or low-intensity activity. This dynamic nature imposes a high physiological demands on athletes, particularly in terms of repeated-effort tolerance and recovery capacity [1,2]. Therefore, the development and maintenance of adequate aerobic capacity represent a key determinant of competitive performance. In addition, several studies have reported significant relationships between body composition and physical performance parameters in soccer players at different competitive levels, including explosive strength, agility, and acceleration, which reinforces the importance of considering both physiological characteristics and nutritional interventions to optimize athletic performance [3].

Among the most widely used indicators to assess aerobic capacity in soccer is maximal aerobic speed (MAS), defined as the minimal running velocity at which maximal oxygen uptake (VO_2_max) is attained. MAS enables the individualization of interval training, optimization of external load prescription, and monitoring of athletes’ functional status [4]. In intermittent sports, its estimation through field-based tests is particularly relevant, as it more accurately reflects the actual demands of match play compared with laboratory-based assessments.

Among specific field-based assessments, the 30–15 Intermittent Fitness Test (30–15 IFT) has been established as a valid, reliable, and sensitive tool for assessing intermittent aerobic fitness in team sports, allowing the detection of meaningful changes in performance [4,5,6,7]. This test combines intermittent running bouts with active recovery periods and incremental speed progression, incorporating changes in direction that simulate the specific demands of soccer [5]. Several studies have demonstrated its usefulness for assessing intermittent aerobic performance, prescribing individualized training loads, and monitoring physiological adaptations in soccer players across different competitive levels [8,9].

In addition, the use of ergogenic aids has gained increasing relevance in different competitive sport contexts, with the aim of optimizing physical and cognitive performance. Among these, caffeine stands out as one of the substances with the strongest scientific support and is classified within Group A by the Australian Institute of Sport [10]. Its primary mechanism of action is associated with the antagonism of adenosine A1 and A2A receptors, which reduces the perception of fatigue, increases alertness, and enhances neuromuscular activation [11,12,13].

Scientific evidence indicates that acute caffeine intake can enhance performance in endurance, power, and high-intensity intermittent exercise, particularly when administered at doses ranging from 3 to 6 mg/kg of body mass [14,15]. In the context of soccer, several studies have reported improvements in repeated sprint ability, high-intensity running distance, and the maintenance of performance during match simulations following caffeine supplementation [16,17].

However, despite the extensive body of evidence on the ergogenic effects of caffeine, most research in intermittent sports has focused on tests such as the Yo-Yo Intermittent Recovery Test or laboratory-based protocols, resulting in limited specific evidence regarding its impact on performance assessed using the 30–15 IFT. This lack of direct studies represents a relevant research gap in applied sport science, particularly in competitive university populations, where training conditions, recovery strategies, and available resources differ from professional settings.

Additionally, factors such as methodological heterogeneity in dosing protocols, administration methods, control of habitual caffeine intake, and individual variations in caffeine metabolism hinder the generalization of previous findings [11,18]. In this regard, there is a need for well-controlled and contextually valid research designs that allow one to assess the true effects of caffeine on specific indicators of intermittent performance. Therefore, the aim of the present study was to analyze the effect of acute caffeine intake on maximal aerobic speed estimated using the 30–15 IFT in university soccer players, employing a randomized, double-blind, crossover design.

## 2. Materials and Methods

### 2.1. Study Design

An experimental, randomized, double-blind, crossover study was conducted to analyze the acute effects of caffeine intake on maximal aerobic speed (MAS) assessed using the 30–15 Intermittent Fitness Test (IFT). Each athlete was evaluated under two experimental conditions (caffeine and placebo), acting as his own control, which allowed for a reduction in interindividual variability and an increase in the statistical power of the analysis. No relevant changes to the protocol were made after trial commencement.

### 2.2. Randomization and Allocation Concealment

Participants were randomly assigned to the caffeine or placebo condition using a computer-generated random sequence (Microsoft Excel RAND function). Randomization was performed by an independent researcher who was not involved in data collection or analysis. Allocation concealment was ensured using sealed, opaque, and sequentially numbered envelopes, opened only at the time of supplementation. The randomization code was kept confidential until the completion of data analysis.

### 2.3. Participants and Setting

Twenty-six male university-level soccer players (age: 24.8 ± 4.6 years) participated in the study. All participants belonged to the university team that won the XXXII ASCUN National University Games in Colombia in 2025. Recruitment was conducted during training sessions through informational meetings. The recruitment and data collection took place in October 2025 in Cali, Colombia. No members of the public were involved in the design, conduct, reporting, or dissemination of this study.

The inclusion criteria were: active membership in the university team, provision of written informed consent, attendance at both experimental sessions, and compliance with pre-test instructions, including abstinence from caffeine for 24 h and avoidance of intense physical exercise for 24 h preceding each trial. The exclusion criteria included: known hypersensitivity to caffeine, history of cardiovascular disease, use of medications affecting caffeine metabolism, habitual caffeine intake exceeding 400 mg/day [11], recent injuries, and any medical condition that could contraindicate participation in the study. Habitual caffeine intake was screened by self-report before enrollment.

### 2.4. Intervention Protocols

The experimental protocol consisted of two testing sessions separated by a 72 h washout period to minimize the residual effects of caffeine. Both sessions were conducted under similar environmental conditions and at comparable times of day. Prior to each session, participants were instructed to maintain adequate hydration, preserve their habitual diet, and abstain from consuming caffeine-containing products for 24 h. Compliance with these instructions was verified through self-report.

Caffeine and placebo capsules were prepared, coded, and distributed by an independent nutrition professional, ensuring proper blinding of the study. Participants ingested either 220 mg of anhydrous caffeine or a placebo capsule (biotin), in accordance with the recommendations of the Australian Institute of Sport (2023), accompanied by water, 45 min before testing. Both participants and researchers were blinded to the assigned condition in each session until the completion of the study.

### 2.5. Measures

Anthropometric characterization was performed following the guidelines of the International Society for the Advancement of Kinanthropometry (ISAK). All measurements were conducted by ISAK Level 2 certified anthropometrists under controlled environmental conditions. Body mass was measured using a calibrated digital scale (Terraillon Fitness Coach Premium; 0–160 kg; accuracy: 100 g), stature with a portable stadiometer (Seca 213; 60–200 cm; accuracy: 1 mm), circumferences with a measuring tape (Lufkin W606PM; 0–200 cm; accuracy: 1 mm), skinfold thickness with a Harpenden caliper (0–75 mm; accuracy: 0.5 mm), and bone diameters with a short anthropometer (Cescorf; 0–164 mm; accuracy: 1 mm). Body fat percentage was estimated using the Yuhasz equation [19] and the sum of six skinfolds, whereas skeletal muscle mass was calculated using the equation proposed by [20].

The 30–15 Intermittent Fitness Test (30–15 IFT) was performed according to the protocol proposed by Buchheit (2008) [5] on an outdoor soccer field. The testing area was marked with cones, establishing two 20 m running zones separated by a central line. The test consisted of 30 s running bouts alternated with 15 s active recovery periods. The initial running speed was set at 8 km/h and increased by 0.5 km/h at each stage, controlled by an auditory signal. The test was terminated when the participant failed to reach the designated line on two consecutive occasions. The final running speed achieved (VIFT) was recorded and used as an estimator of maximal aerobic speed (MAS).

Supplementation consisted of a fixed dose of 220 mg of anhydrous caffeine (commercially available anhydrous caffeine capsules), selected based on safety recommendations and previous studies reporting ergogenic effects with standardized doses, in accordance with the Australian Institute of Sport (2023). Based on participants’ body mass, the relative caffeine dose corresponded to 2.85 ± 0.27 mg/kg (range: 2.60–3.16 mg/kg). The placebo consisted of biotin capsules which were identical in size, shape, and color, and were administered with approximately 250 mL of water. Biotin was selected as a placebo because it is not expected to exert acute ergogenic effects at the administered dose and allowed identical capsule appearance to maintain blinding. Participants were monitored for potential adverse effects during and after testing, and no clinically relevant events were recorded. Potential adverse effects related to caffeine intake were assessed using a standardized self-report questionnaire administered before supplementation and testing sessions. Participants were instructed to report any discomfort, symptoms, or unusual sensations experienced during or after the intervention. This relative dose was located at the lower limit of the ergogenic range typically reported in the literature (3–6 mg/kg).

### 2.6. Sample Size Determination

The sample size was determined by the availability of the competitive roster and the crossover design. This design reduces interindividual variability and increases statistical efficiency for detecting acute supplementation effects. In addition, previous studies on caffeine and intermittent performance have reported moderate-to-large effects, supporting the theory that a sample size in the range of typical team-based crossover trials is adequate for detecting meaningful differences [11,14]. A formal a priori power calculation was not conducted due to the team-based nature of participant recruitment.

### 2.7. Statistical Analysis

Data analysis was performed using SPSS software (IBM Corporation, USA), version 26.0 for Mac. Variables were described using means and standard deviations (SD). Data normality was assessed using the Shapiro–Wilk test. Comparisons between the caffeine and placebo conditions were conducted using paired Student’s *t*-tests. Effect size was calculated using Cohen’s d (dz) and interpreted as small (0.20–0.49), moderate (0.50–0.79), or large (≥0.80) [21]. Ninety-five percent confidence intervals (95% CI) were calculated for mean differences and effect size estimates to improve statistical precision. The level of statistical significance was set at *p* < 0.05. In addition, absolute and relative differences (%) were calculated to assess the practical relevance of the results.

### 2.8. Ethics Approval

This study was conducted in accordance with the principles and procedures established to ensure the protection of human participants, in compliance with the Declaration of Helsinki [22] and Resolution 8430 of 1993 of the Colombian Ministry of Health on personal data protection. The research protocol was approved by the Ethics Committee of the Institución Universitaria Escuela Nacional del Deporte, Cali, Colombia, under approval number 320.02-08-2025, dated 7 September 2025.

All participants provided written informed consent prior to their inclusion in the study. Confidentiality, data anonymity, and the right to withdraw voluntarily at any time were guaranteed in accordance with the approval granted by the Ethics Committee.

## 3. Results

All 26 participants completed both experimental conditions and were included in the final analysis. No participants withdrew from the study, and no data were excluded after randomization. Anthropometric characterization allowed the establishment of the morphological profile of the sample (*n* = 26). The players presented values consistent with trained university populations, characterized by a low body fat percentage and high muscle mass. Table 1 shows the mean age was 24.8 ± 4.6 years, with an average stature of 178.9 ± 6.0 cm and a body mass of 77.1 ± 7.5 kg.

Body mass index (BMI) was 24.1 ± 1.8 kg/m^2^. The body fat percentage estimated using the Yuhasz equation was 12.5 ± 2.9%, whereas the skeletal muscle mass calculated using the Lee equation was 41.3 ± 3.6 kg. These results indicate an appropriate body profile for performance in intermittent sports such as soccer.

Under the placebo condition, participants completed the 30–15 IFT without ergogenic intervention. The final running speed (VIFT), used as an estimator of maximal aerobic speed, showed a mean value of 18.7 ± 1.5 km/h in Table 2, with values ranging from 16.5 to 21.0 km/h. These results represent the group’s baseline level of intermittent aerobic performance and served as a reference for comparative analysis. In contrast, under the caffeine supplementation condition (220 mg), a generalized improvement in performance was observed. The mean VIFT increased to 19.9 ± 1.7 km/h, indicating a clear enhancement compared with the placebo condition. Most players exhibited individual increases in final running speed, suggesting a consistent ergogenic response to the intervention.

Statistical analysis, as shown in Table 3, revealed significant differences between the caffeine and placebo conditions. The paired Student’s *t*-test showed a value of t = 5.25, with a significance level of *p* < 0.001. The mean absolute difference was 1.22 km/h, representing a relative improvement of 6.5% in performance under the caffeine condition. The effect size was large (dz = 1.24; 95% CI: 0.65–1.82), indicating the substantial magnitude of the supplementation effect.

These results indicate that acute caffeine intake was associated with a significant increase in final running speed during the 30–15 Intermittent Fitness Test.

## 4. Discussion

The aim of the present study was to analyze the effect of acute caffeine intake on maximal aerobic speed estimated using the 30–15 IFT in university soccer players. The main findings indicate that supplementation with 220 mg of caffeine was associated with a significant improvement in intermittent aerobic performance, reflected by an average increase of 1.22 km/h (6.5%) in final running speed, accompanied by a large effect size (dz = 1.24). These results support the proposed hypothesis and indicate a meaningful ergogenic effect in this context, although the magnitude of the effect may be influenced by sample characteristics and study design.

The performance values obtained under the placebo condition (18.7 ± 1.5 km/h) fall within the ranges reported for university and semi-professional soccer players, suggesting that the sample presented an adequate level of physical fitness. In addition, the anthropometric profile characterized by a low body fat percentage (12.5%) and high muscle mass (41.3 kg) is consistent with previous studies identifying these characteristics as factors associated with greater tolerance to intermittent exercise and improved running economy [23,24]. Furthermore, evidence indicates that body composition, particularly a lower fat percentage and higher levels of explosive strength, is related to superior performance in sprinting, jumping, and agility tests in semi-professional soccer players, which complements the ergogenic effects observed following caffeine intake on maximal aerobic speed [3].

The magnitude of improvement observed following caffeine intake falls within the range reported in systematic reviews and meta-analyses, which describe increases of 2% to 8% in endurance performance and high-intensity intermittent exercise [14,15]. In the specific context of soccer, Lara et al. (2020) [16] and McLellan et al. (2016) [17] reported improvements in repeated sprint ability, maintenance of exercise intensity, and high-speed running distance, findings that are consistent with those of the present study.

An important aspect of the present study is that a fixed dose of caffeine (220 mg) was used, corresponding to approximately 2.8 mg/kg of body mass based on the average body mass of the sample. This relative dose is located at the lower limit of the conventionally recommended ergogenic range (3–6 mg/kg); however, it was sufficient to produce a meaningful improvement in intermittent aerobic performance. These findings are consistent with the recommendations of the Australian Institute of Sport (2023), which recognize the effectiveness of standardized doses of at least 200 mg in applied sport settings, where precise individualization based on body mass may not always be feasible.

The use of a fixed absolute dose likely introduced some degree of interindividual variability in relative caffeine intake, as each participant received a slightly different dose when expressed relative to body mass. This variability may partially explain differences in individual responsiveness and should be considered when interpreting the magnitude of the ergogenic effect.

The observed effect size (dz = 1.24) was higher than that reported in many previous studies, in which small-to-moderate effects were commonly observed, and should therefore be interpreted with caution. This difference may be partially explained by the homogeneity of the sample in terms of age, training level, and anthropometric characteristics. The reduction in interindividual variability enhances the sensitivity of the crossover design to detect intervention-induced changes, particularly in highly reproducible tests such as the 30–15 IFT [4].

From a physiological perspective, the mechanisms underlying these results may be related to the antagonism of adenosine receptors, increased catecholamine release, and reduced perception of effort, which may promote greater fatigue tolerance and more efficient neuromuscular activation [11,12,13]. In intermittent exercise, these effects may translate into an enhanced ability to sustain high running speeds over prolonged periods, as reflected by performance during the 30–15 IFT. However, the present study was not designed to directly assess physiological mechanisms, and therefore these interpretations should be considered theoretical and based on existing literature.

Additionally, the specific nature of the 30–15 IFT may have enhanced the sensitivity of the protocol to the effects of caffeine. By incorporating changes in direction, active recovery periods, and incremental speed progression, this test closely reproduces the physiological demands of soccer, thereby facilitating the transfer of ergogenic benefits to real competitive situations [5].

From an applied perspective, the present results suggest that caffeine may represent a potentially useful strategy for supporting intermittent aerobic performance in university soccer players under similar applied conditions, particularly in competitive contexts or physical performance assessments. However, its implementation should be individualized, taking into account factors such as tolerance, habitual intake, chronotype, and potential sleep disturbances, as highlighted by Grgic et al. (2020) [14] and Guest et al. (2021) [11]. Furthermore, training-induced improvements in strength and speed should also be considered, as they contribute to performance optimization and illustrate how multiple strategies, including nutrition and training, may interact to influence performance outcomes [25]. These findings should be generalized cautiously and primarily to populations with similar characteristics, training status, and competitive level.

Among the main limitations of this study are the relatively small sample size, which is characteristic of research conducted in university sport settings and should be considered when interpreting the magnitude of the findings, as well as the absence of genetic control of caffeine metabolism (CYP1A2 polymorphism). In addition, complementary variables such as perceived exertion, heart rate, and cognitive indicators, which could have provided a more comprehensive understanding of the response to supplementation, were not assessed.

Future studies should consider multicenter designs, compare different body mass–adjusted doses, include female populations, and analyze additional physiological and perceptual variables. In this way, further evidence can be generated to strengthen the strategic use of caffeine in intermittent sports. No additional subgroup or sensitivity analyses were performed.

## 5. Conclusions

Acute intake of 220 mg of caffeine was associated with a significant improvement in intermittent aerobic performance, reflected by an average increase of 1.22 km/h in final running speed, equivalent to a relative improvement of 6.5%, with a large effect size. These findings suggest that acute caffeine supplementation may serve as an ergogenic aid for improving intermittent aerobic performance in university soccer players under the conditions of the present study, even when moderate standardized doses are employed. Therefore, the controlled use of caffeine could be considered as a potentially practical and accessible strategy in similar applied contexts, provided that individual biological characteristics and tolerance are taken into account. Overall, the findings should be interpreted within the specific methodological context of the study.

## Figures and Tables

**Table 1:** Body Composition (*n* = 26).

Variable	Mean ± SD	CI (95%)	Normality
Age (years)	24.8 ± 4.6	22.4–27.1	0.689
Stature (cm)	178.9 ± 6.0	175.9–181.9	0.962
Body mass (kg)	77.1 ± 7.5	73.4–80.8	0.180
BMI (kg/m^2^)	24.1 ± 1.8	23.2–25.0	0.221
Body fat (%)	12.5 ± 2.9	11.0–13.9	0.189
Σ6 skinfolds (mm)	57.5 ± 18.7	48.2–66.8	0.189
Skeletal muscle mass (kg)	41.3 ± 3.6	39.5–43.1	0.141

SD: Standard deviation; CI: Confidence interval for the mean; Normality test: Shapiro–Wilk.

**Table 2:** 30–15 IFT Result Placebo and Caffeine Conditions (*n* = 26).

Variable	Mean ± SD	CI (95%)	Normality
VIFT Placebo (km/h)	18.7 ± 1.5	18.0–19.5	0.319
VIFT Caffeine (km/h)	19.9 ± 1.7	19.1–20.8	0.389

SD: Standard deviation; CI: Confidence interval for the mean; Normality test: Shapiro–Wilk.

**Table 3:** Comparison of 30–15 IFT Performance (*n* = 26).

**Caffeine vs. Placebo**	**Dif. (km/h)**	**% Imp**	**t**	***p*** **-Value**	**dz**	**95% CI**
	+1.22	6.5%	5.25	*p* < 0.001	1.24	0.65–1.82

Dif: Difference (km/h); Imp: Improvement; t = *t*-value; dz: Cohen’s Effect size.

## Data Availability

The data presented in this study are available upon request from the corresponding author.
